# Wind-Sifting Separation: A Review

**DOI:** 10.1021/acsomega.3c01087

**Published:** 2023-09-12

**Authors:** Jimmy Alade, Samson Oluwaseyi Bada

**Affiliations:** DSI-NRF SARChI Clean Coal Technology Research Group, Faculty of Engineering and the Built Environment, University of the Witwatersrand, Wits 2050, Johannesburg, South Africa; University of the Witwatersrand, Wits 2050, Johannesburg, South Africa

## Abstract

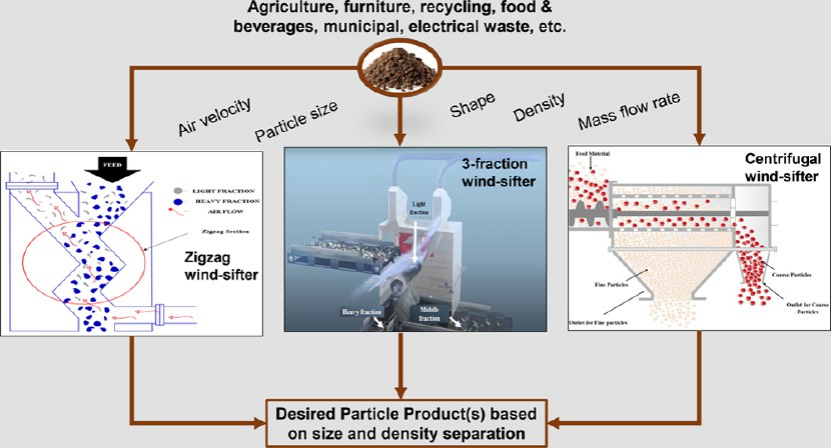

Dry particle classification is a viable alternative to wet classification, both financially and environmentally, and has been used for decades with several approaches and techniques. One of these techniques, the wind-sifting principle, has been observed to be very effective for particle separation. Its separation mode is based on the use of the physical properties of these particles such as size, shape, and density to carry out separation. The principle of wind-sifting has been used to design multiple separators with various configurations for diverse kinds of applications, including recycling, agriculture, furniture, food and beverages, municipal and electronic waste sorting, and even mineral-processing industries. Although the wind-sifting principle has been implemented for various applications, research of this principle is ongoing owing to minimal literature. This Review seeks to provide some literature on wind-sifters as it delves into the three main types, their generic design features, and operational principles.

## 1 Introduction

The concept of particle sorting is essentially a method of separating mineral particle mixtures into two or more particles based on the continuous decline in the velocity with which the traveling particles fall through a fluid medium.^1^ This concept is well used in numerous industries, including agriculture, food processing, and, in particular, mineral processing. The classification of the particles is made possible by the physical properties of the particles such as their shape, size, and density. Solid particles falling freely in a vacuum are subject to constant acceleration as their velocity increases indefinitely, regardless of size and density. However, in air or water, there is a resistance to this movement because of viscosity. Resistance increases with velocity, and when equilibrium is attained between the gravitational and fluid resistances forces, the particles come to rest as they attain what is known as the terminal/settling velocity.^1,2^ Particle separation is possible when these solid particles reach their respective settling velocities due to different sizes and densities.

There are two types of particle classifications which are based on the fluid used, the wet classification and the dry classification. The wet classification entails the use of hydraulic fluids (liquids) like water for the separation of particles. While this classification is known to provide excellent separation efficiency, some drawbacks, including negative environmental impacts and high operational capital costs, have made dry classification a viable alternative.^1−3^ The primary purpose of classification is to separate coarse particles in a particle mix from fine particles. With dry classification, the processing of particles in various sizes ranging from fine powder to pellets, flakes, and even two-dimensional materials such as paper and aluminum foil is achievable.^1,4^ The separation of particles without using water as a sorting medium has been implemented for decades, with many new inventions and techniques now in the literature. Some of these techniques involve ballistic air separators, winnowing separators, and wind-sifting separators, among others.^5−8^

In the case of wind-sifting, this technique sometimes involves the use of sieves for the classification of particles and sometimes without sieves, as seen with the zigzag wind-sifter, which uses air primarily for particle sorting.^9^ The wind-sifting separation techniques have been used in various applications and in the diverse recycling of materials like electronic waste (e-wastes), municipal solid wastes, food, furniture, pharmaceuticals, minerals, chemicals, and metallurgical, and it has recently been applied in the field of coal processing.^2,9,10^

Hagemeier et al.^11^ used computational fluid dynamics simulations and discrete particle modeling in a coupled manner to carry out a numerical investigation on the separation efficiency of a pilot-scale zigzag wind-sifter separator. The influence of process parameters such as airflow velocity and particle size was investigated under various turbulence models. The results showed that process performance varied with changes in process parameters. The author also observed that residence times for light particles were much longer than for heavy particles. This results from the lighter particles easily being influenced by the slight change in air velocity, compared to the heavier particles, making them easily affected by the vortices, thus increasing their residence time.

In a study conducted by Mann et al.,^12^ the zigzag wind-sifting technique was seen to be very suitable in the processing of raw agricultural materials and their products. Roloff et al.^13^ conducted a study where a multicamera shadow-imaging system was installed in a wind-sifter separator to capture the dynamics of the particles in the separator. The fabricated wind-sifter separator was used to separate glass beads of 1–4 mm at different particle loads (mass flow rates) as well as air velocities. The results revealed that the particle air velocity has more influence on the separation efficiency of the separator compared to the particle loading for the classification of the particles. Reddy et al.^14^ also used the zigzag wind-sifter separator for the separation of food particles such as milk, spices, starch, sugar, salt, flour, and grain. With the application of this technique, the high capital and operating expenses that would have been encountered with the use of sieves to separate these food particles were circumvented.

The most recent wind-sifter separator was fabricated by Alade et al.;^2^ the separator was used by the author to upgrade run-of-mine (ROM) coal from the Witbank coalfield, South Africa. The result obtained shows that the separator was effective in upgrading a feed coal of 30.28% ash content and 21 MJ/kg calorific value to a clean coal of 18.94% ash content with a calorific value of 26.8 MJ/kg. This was the first time that this technology was applied in the field of dry coal beneficiation and served as a prototype, with an optimized version in view. Although various configurations of the wind-sifter separators exist, the three major types of wind-sifters are the zigzag sifters, the rotary/drum/centrifugal sifters, and the 3-fraction wind-sifters as well as some other innovative approaches for wind-sifting. This Review aims to give a bolder outlook on the three major types of wind-sifter separators, their design features, their modes of operations, and their feasibility in the processing of various minerals other than coal. In addition, the article also looks at some recent innovative approaches of using the wind-sifting principle for particle separation.

## 2 Wind-Sifting

### 2.1 The Zigzag Wind-Sifter

#### 2.1.1 General Design Features and Mode of Operation

**Figure 1:**
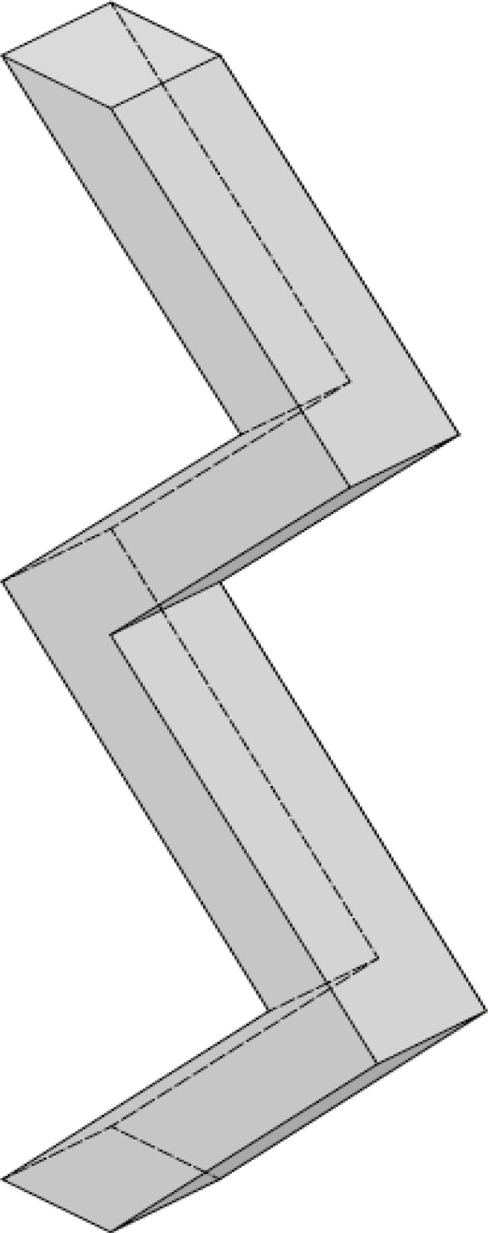
General design of the zigzag section.

The zigzag wind-sifter is a special wind-sifter separator which consists of cascading connected vertical channels. The channels alternatively slant left and right and facilitate the separation of a solid dispersed particle phase into its light fine and heavy coarse fractions.^14^ The zigzag sifter is the most common type of sifting separation technique used and is used for a variety of applications such as waste processing and recycling, agricultural, furniture, mineral processing, etc. The main component of this kind of wind-sifter is its zigzag section, from where its name is derived. Figure 1 provides a general representation of the segmented section in question.

**Figure 2:**
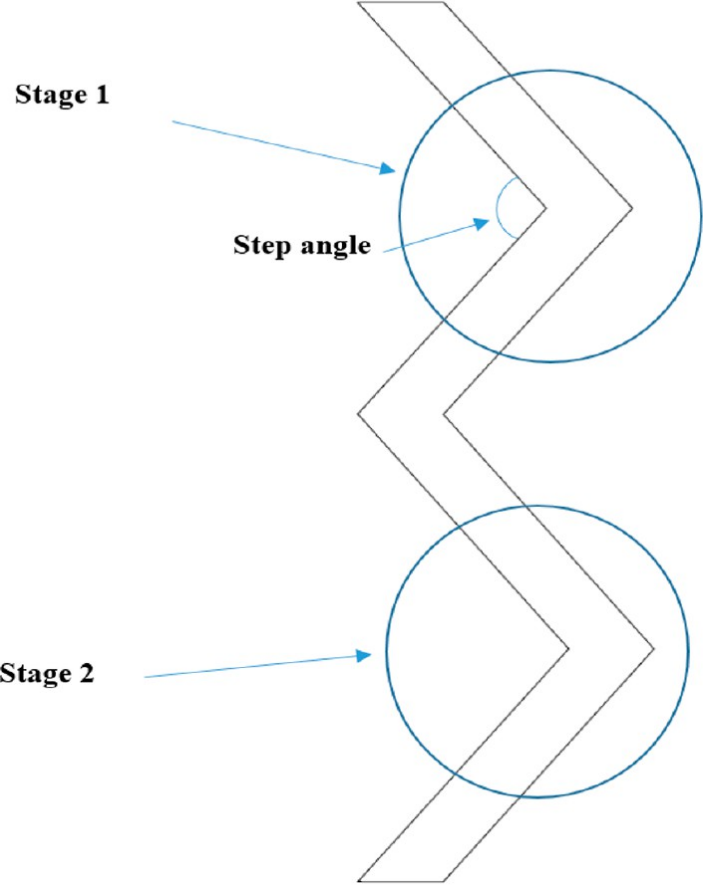
Side view of the zigzag wind-sifter, adapted from Kaas et al.^3^

Kaas et al.^3^ gave an elucidating review of the zigzag sifter where they outlined design parameters of the zigzag section, some of which include stages and step angles. Each stage is the amount of the segmented zigzag cross sections, while the step angle of the section in each stage is as shown in Figure 2. The separator overall design can vary based on the manufacturer’s design like Rusmagnet,^15^ Impact Air Systems,^16^ Trennso-Technik,^17^ etc., for their respective applications. In the Kaas et al.^3^ design, the step angle for each stage was set to 120°. This was to ensure an adequate amount of kinetic energy in the sifting for the transport of the lighter particles.

#### 2.1.2 Operational Principle of the Zigzag Sifter

**Figure 3:**
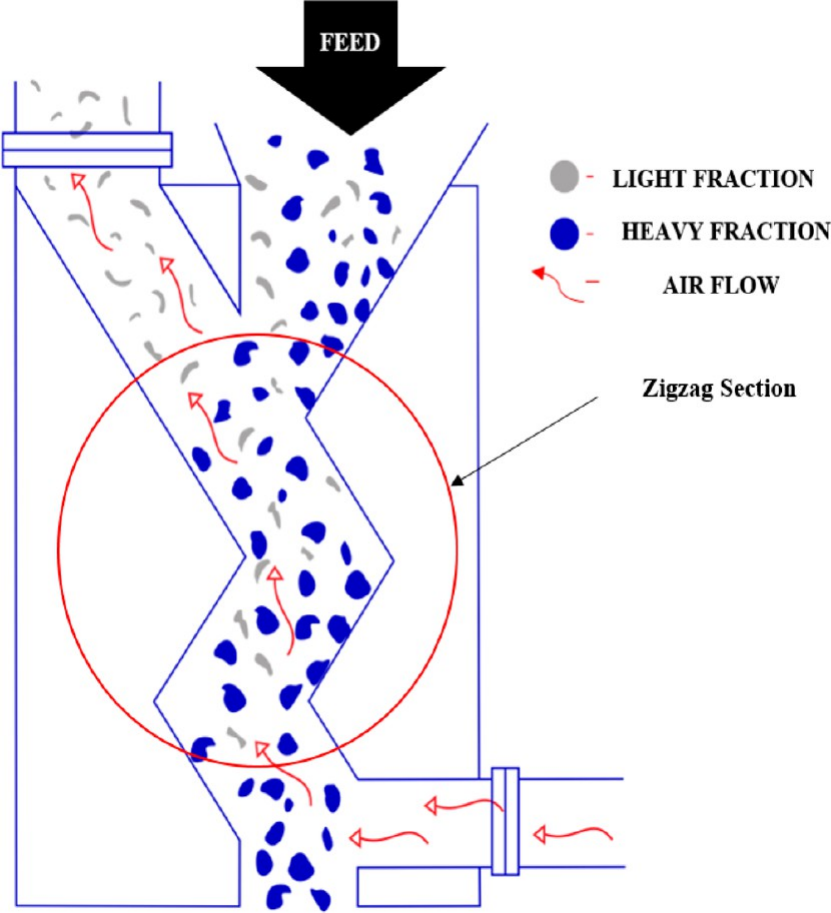
Working principle of the zigzag separator, image courtesy of ERGA Global.^15^

**Figure 4:**
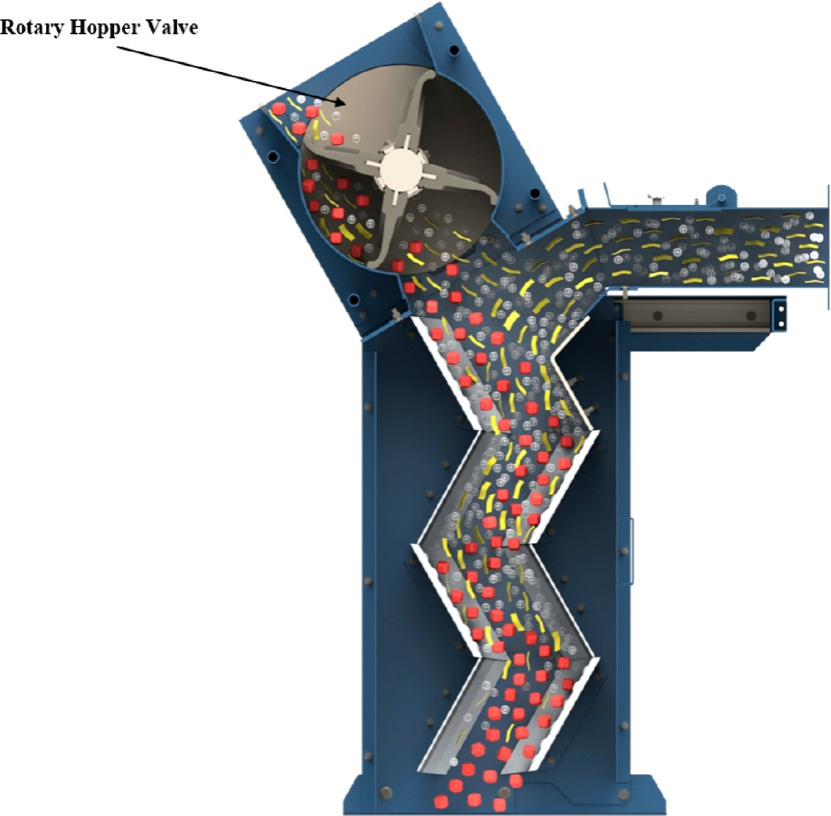
Image showing the rotary hopper valve, adapted from Impact Air Systems.^16^

Figure 3 shows the basic operation of a zigzag wind-sifter separator. Air is supplied by a mechanical draft (forced draft), usually with the aid of a blower from the bottom of the zigzag section of the separator, as shown in Figure 3. The feed material to be separated is fed directly into the zigzag separator usually with the aid of a rotary valve, as indicated in Figure 4. This is then distributed over the complete sifter channel cross section. In the zigzag section, the feed materials bounce through this section as they cascade down the stage(s), with the heavier particles bouncing more than the lighter particles. This is due to the incoming air supply sifts, leading to the transport of the lighter particles further while the heavier particles fall into their designated collection zone.

**Figure 5:**
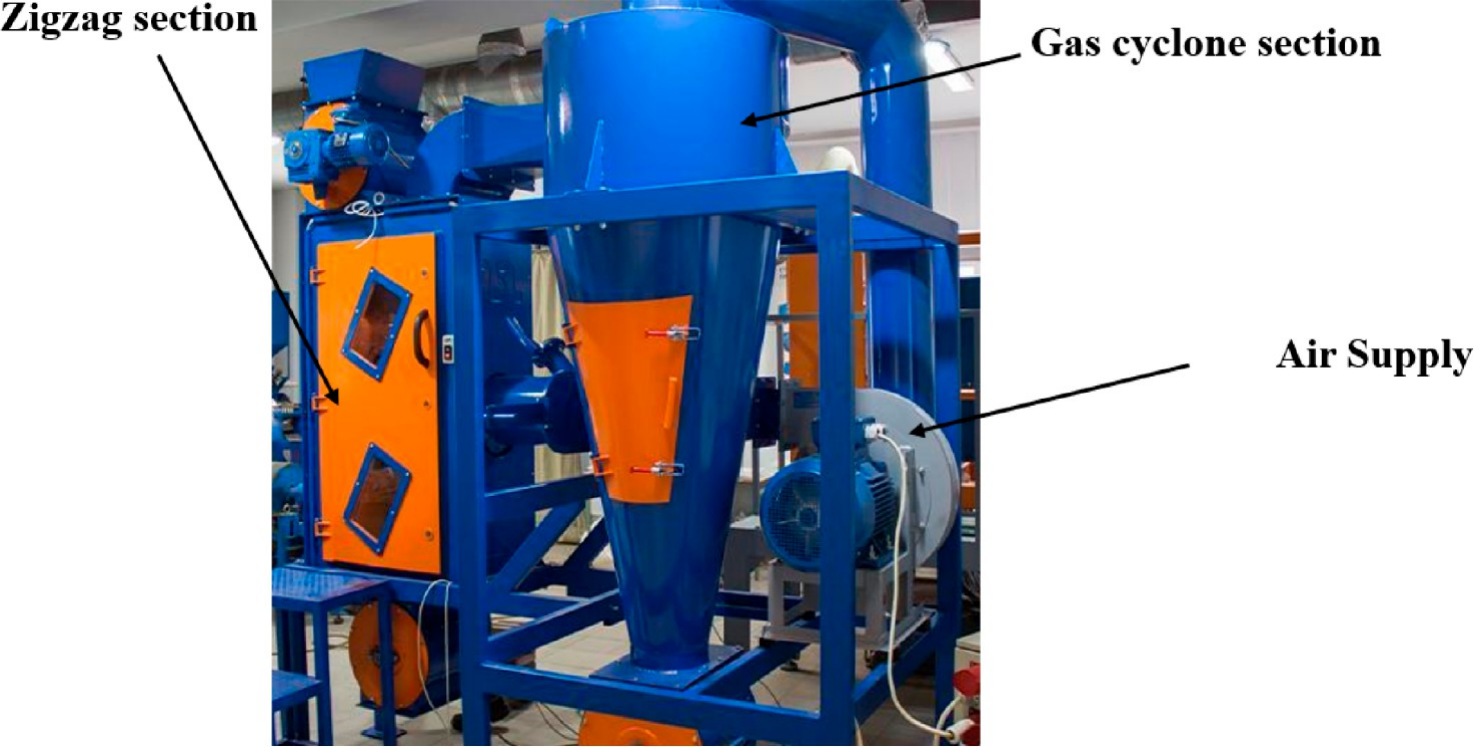
Zigzag separator with a gas cyclone, image courtesy of ERGA.^15^

Generally, the zigzag sifter separator is used alongside other equipment, rather as a stand-alone unit. This is because the settling velocities of the lighter particle being separated from the heavier ones is not attained, even though separation took place in the separator. This type of wind-sifter engages both the countercurrent and cocurrent modes of particle separation. The countercurrent separation occurs when the incoming air meets with the incoming feed, while with the cocurrent separation the lighter particles are transported to their settling zones. Major companies like Impact Air Systems usually combine their zigzag separators with gas cyclone equipment to ensure adequate classification. Some researchers have combined the zigzag separator with other separators like the ballistic air separator depending on the nature of the application like municipal solid wastes and electronic waste treatment.^15−17^Figure 5 shows a typical zigzag sifter and gas cyclone assembly.

**Figure 6:**
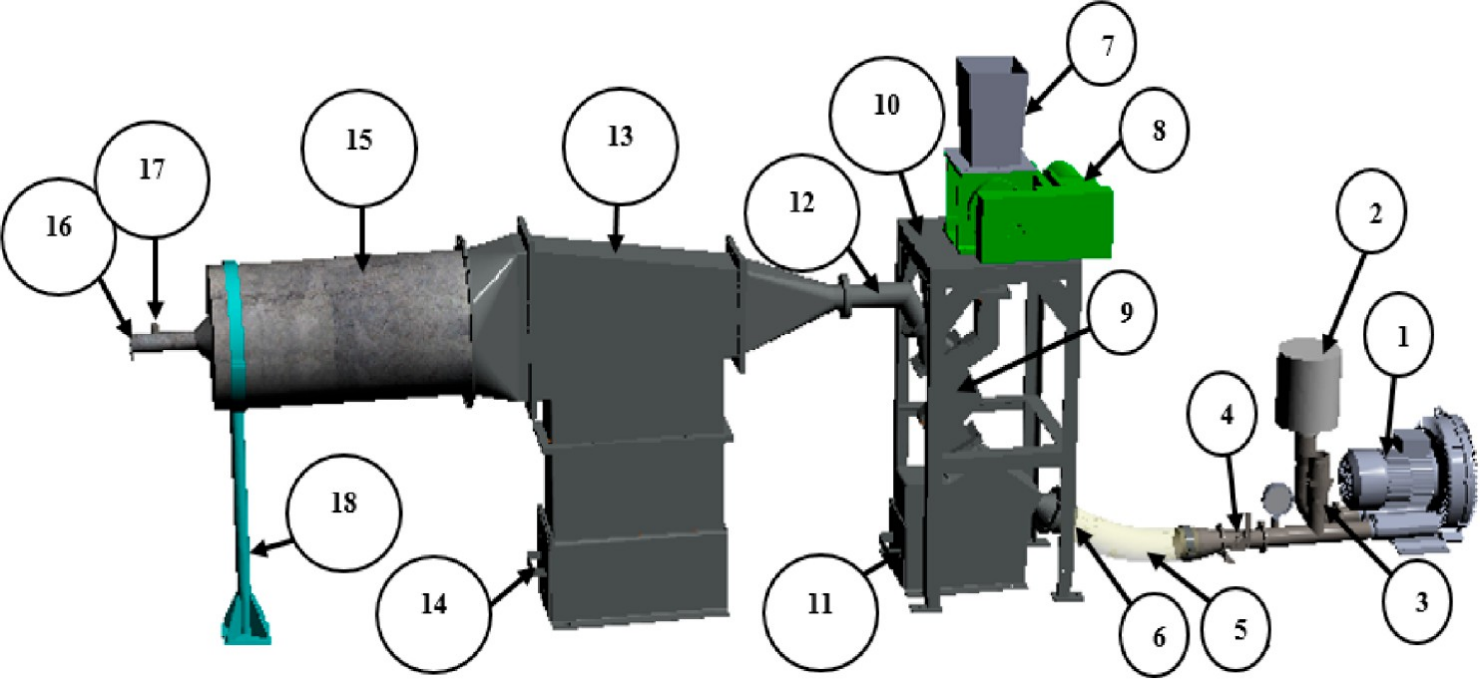
Designed zigzag separator for dry coal beneficiation by Alade et al.^2^. 1, air blower; 2, air filter; 3, safety relief valve; 4, throttle damper; 5, 100 mm diameter flex; 6, air inlet into the first chamber; 7, rotary airlock valve for the coal-feed; 8, variable frequency drive motor drive for the rotary hopper valve; 9, first chamber (the wind-sifter) with the zigzag section; 10, support frame for the wind-sifter; 11, coal bin for the first chamber; 12, exit of the clean coal from the first chamber into the second chamber; 13, second chamber (diffuser chamber) to collect the clean coal; 14, coal bin for the second chamber; 15, filter section (with filter cartridge) to prevent coal dust from escaping to the environment; 16, air exit from the separator; 17, velocity measuring port; 18, support stand for the filter compartment.

The research conducted by Alade et al.^2^ for dry coal beneficiation entailed the use of the zigzag separator in tandem with a diffuser separation chamber (cocurrent separation). The function of the diffuser chamber is to ensure clean coal products are separated based on their relative densities (the cleaner the coal, the further the particles travel to their settling points). The schematic of the separator designed by the author is illustrated in Figure 6, which demonstrates that the zigzag separator is very flexible. It cut sizes and densities through the changes in process parameters such as particle loading/feed rate as well as the airflow rate into the separator.^2,3,15−17^

### 2.2 Rotary Centrifugal Wind-Sifters

#### 2.2.1 General Design Features and Mode of Operation

**Figure 7:**
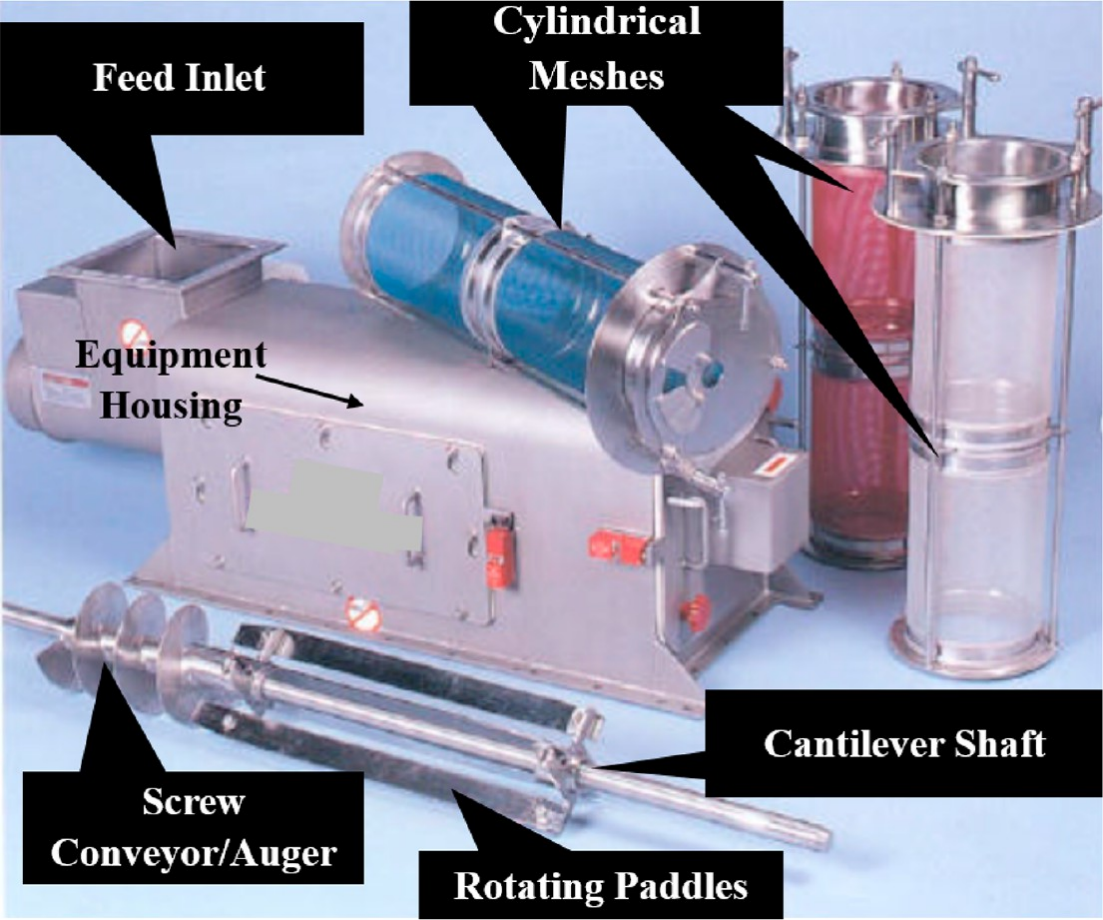
Major components of a centrifugal sifter, image courtesy of Gericke.^19^

The rotary centrifugal sifter is not as common as the zigzag sifter and is usually used to process a wide range of free-flowing materials such as powders, agglomerates, spices, turmeric, cosmetics, pharmaceuticals, chemicals, minerals, fibrous material, sawdust, coconut shells, tobacco, and granules.^18−20^ The design works primarily on the principle of applying centrifugal force to the feed particles. Major components of this sifter can be seen in Figure 7, including a feed inlet, a cylindrical mesh or screen, rotating paddles, a cantilever shaft driven by an electric motor, and a screw conveyor which is a screw wound around the shaft in a helix form, also called an auger.

#### 2.2.2 Operational Principle of the Rotary/Centrifugal Sifter

**Figure 8:**
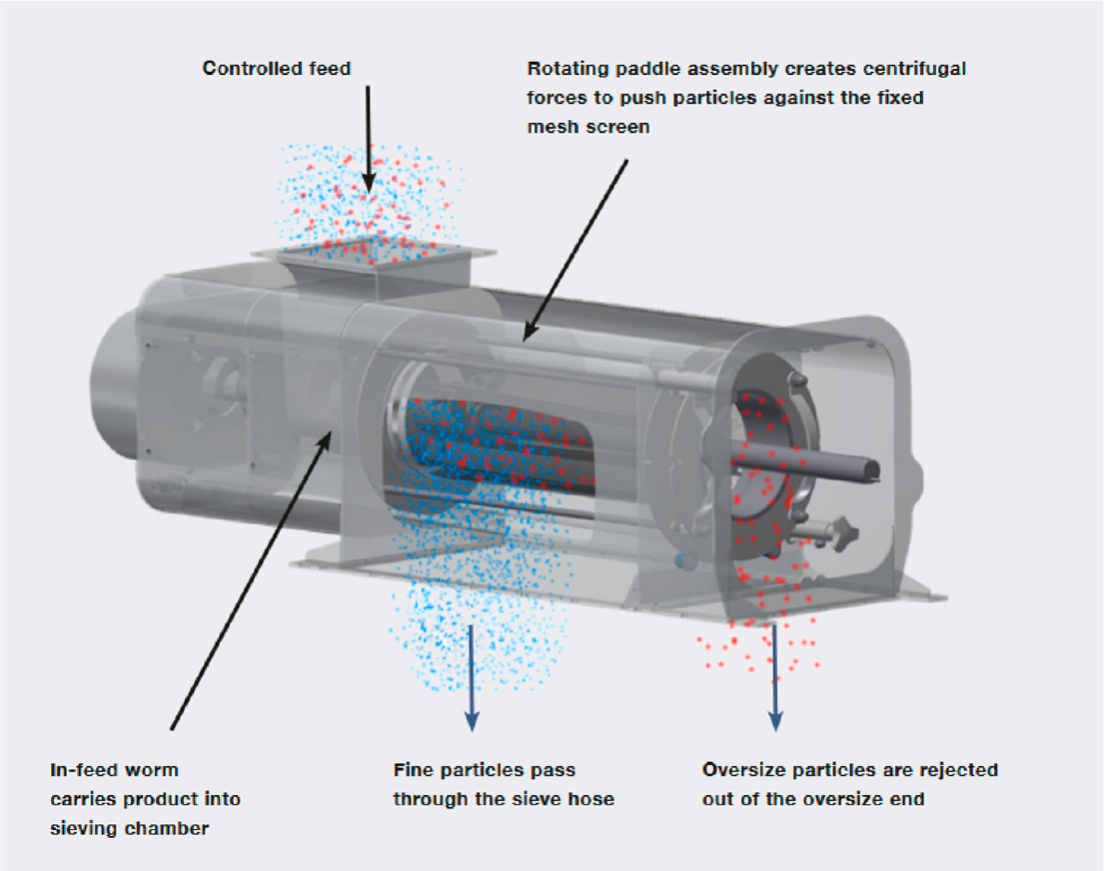
Side view of a centrifugal sifter, image courtesy of Gericke.^19^

In operation, the feed material to be separated is fed in through the feed inlet which is at the upper section of the housing and the rotating paddles (also known rotating scrappers). The paddles transport the feed material into the mesh section of the centrifugal sifter by centrifugal force and cyclone propelling action.^18,19^Figure 8 gives an illustration of the flow path of the feed material through the separator.

**Figure 9:**
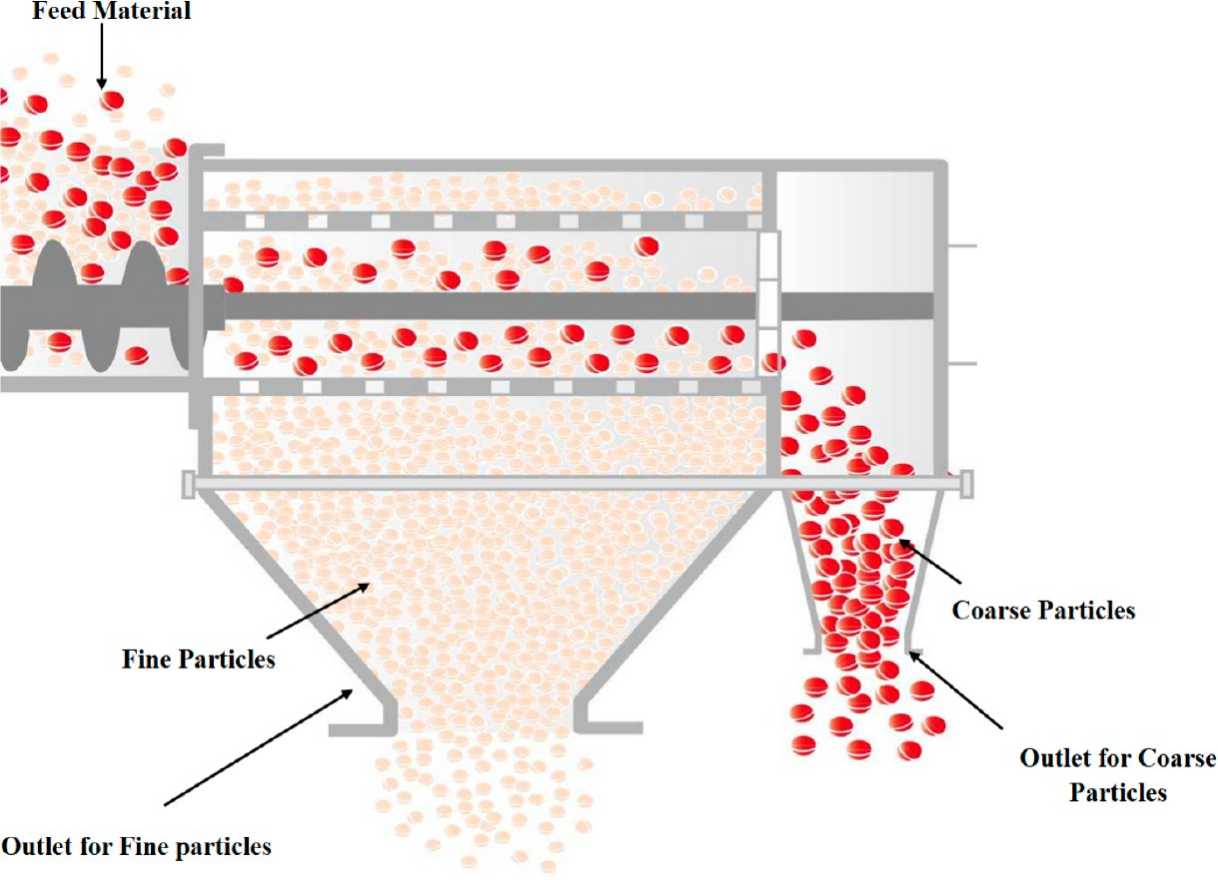
Side view of a centrifugal sifter, image adapted from Gericke.^19^

**Figure 10:**
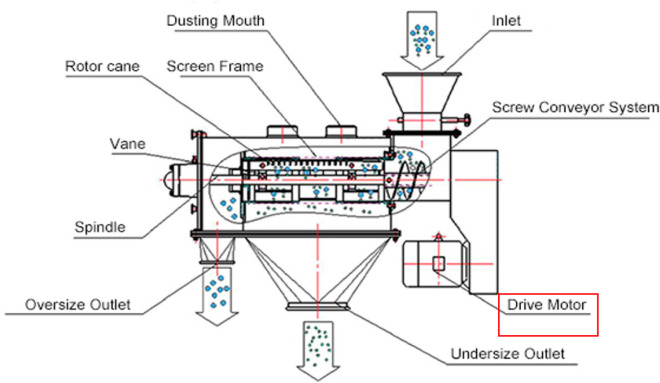
Closed centrifugal sifter, image courtesy of Shree Bhagwati Group of Companies.^18^

The finer particles pass through the cylindrical mesh while the coarser particles which cannot pass through the mesh continue moving forward as they are being transported by the rotating paddles. Figure 9 displays the respective outlets for both the fine and coarse particles in a typical centrifugal sifter, while Figure 10 shows a side-view schematic of the centrifugal sifter when closed. It is important to note that the air in this wind-sifter is generated by the rotating action of the paddles that are coupled to the cantilever shaft and not an external air supply. This contrasts with the zigzag wind-sifter, which mainly uses a cocurrent airflow. Also, this sifting classifies particles mainly on size as opposed to the zigzag sifter which classifies on size, shape, and density.

**Figure 11:**
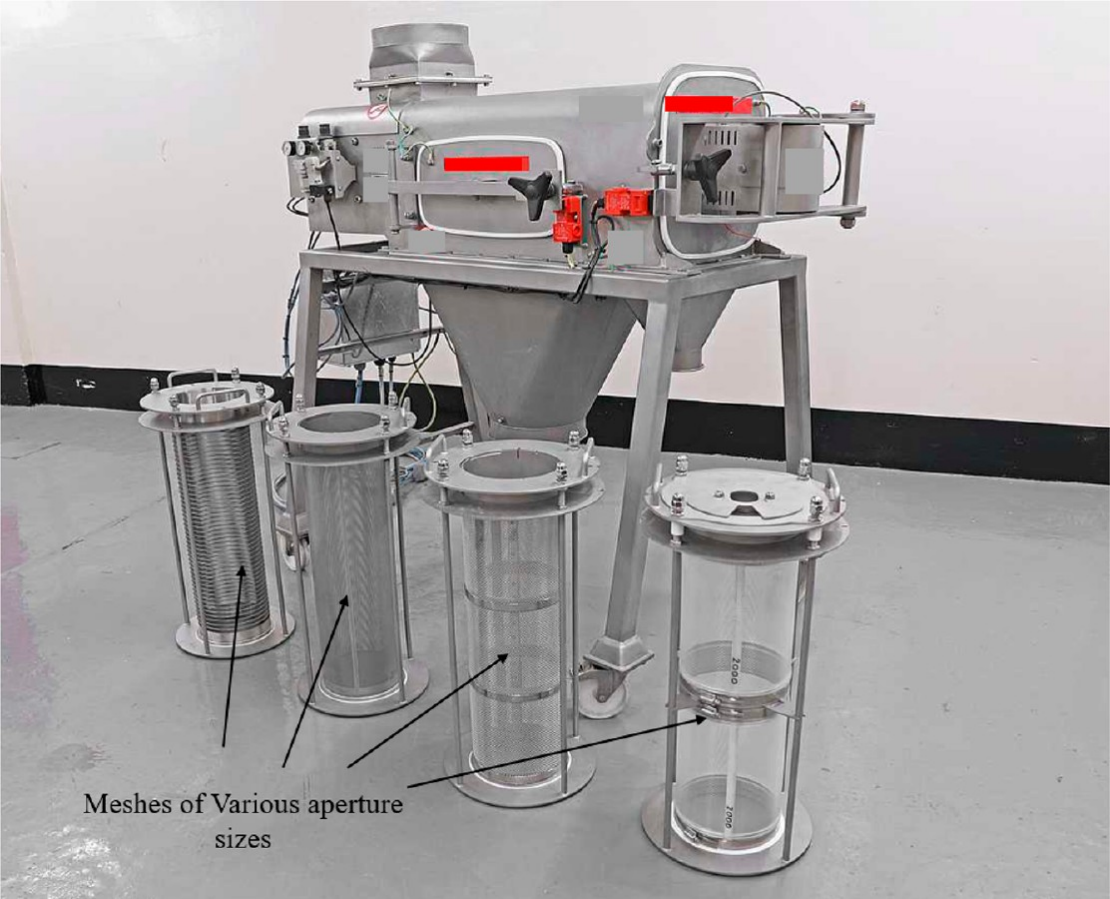
Mesh of different aperture sizes, image courtesy of and adapted from Gericke.^19^

The flexibility of this sifting process depends on the ability to change the cylindrical mesh to different aperture sizes to obtain a product of different qualities. Figure 11 shows different cylindrical meshes of various aperture sizes.

### 2.3 The 3-Fraction Wind-Sifter

The 3-fraction wind-sifting is a relatively new technique compared to zigzag wind-sifting and is preferably used by waste companies owing to its high efficiency, to process heterogeneous municipal solid waste materials into three fractions.^21^ In this technique, an air wheel is used to partition the airstream which is transporting the particles to heavy, medium, and light fractions at the operator’s discretion.

#### 2.3.1 General Design Features and Mode of Operation

**Figure 12:**
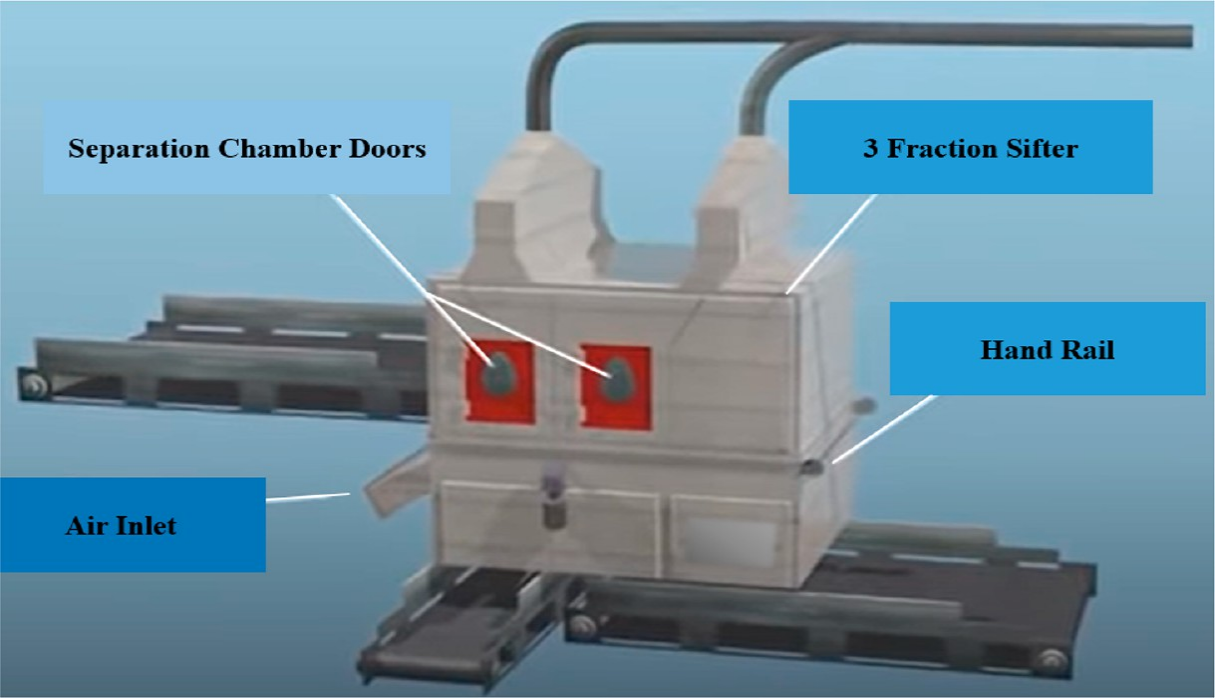
Three-fraction Sifter, image courtesy of and adapted from Schulz and Berger.^21^

**Figure 13:**
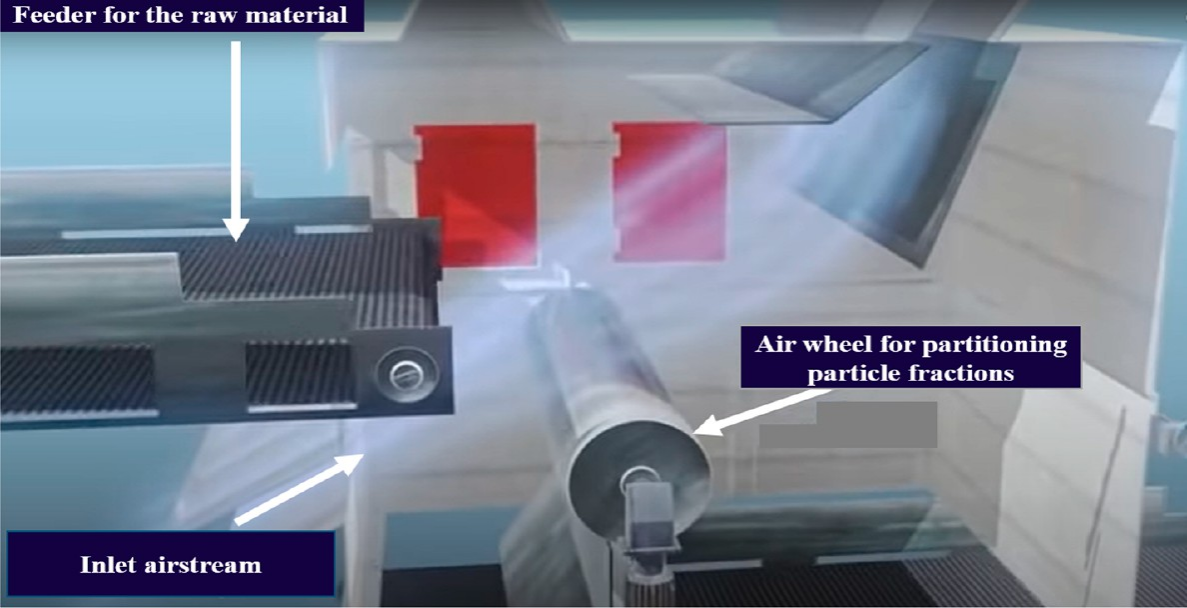
Image showing inlet air and air wheel, Image courtesy of and adapted from Schulz and Berger.^21^

The 3-fraction wind-sifter has an adjustable vertex drive shaft, which can be adapted for various purposes, to control the airflow velocity which is the primary medium for controlling the feed material. Major components of this sifter include the air-inlet section separation chamber doors, handrails as can be seen in Figures 12, and a feeder for the raw materials, as well as an air wheel, which can be seen in Figure 13.

#### 2.3.2 Operational Principle of the 3-Fraction Wind-Sifter

**Figure 14:**
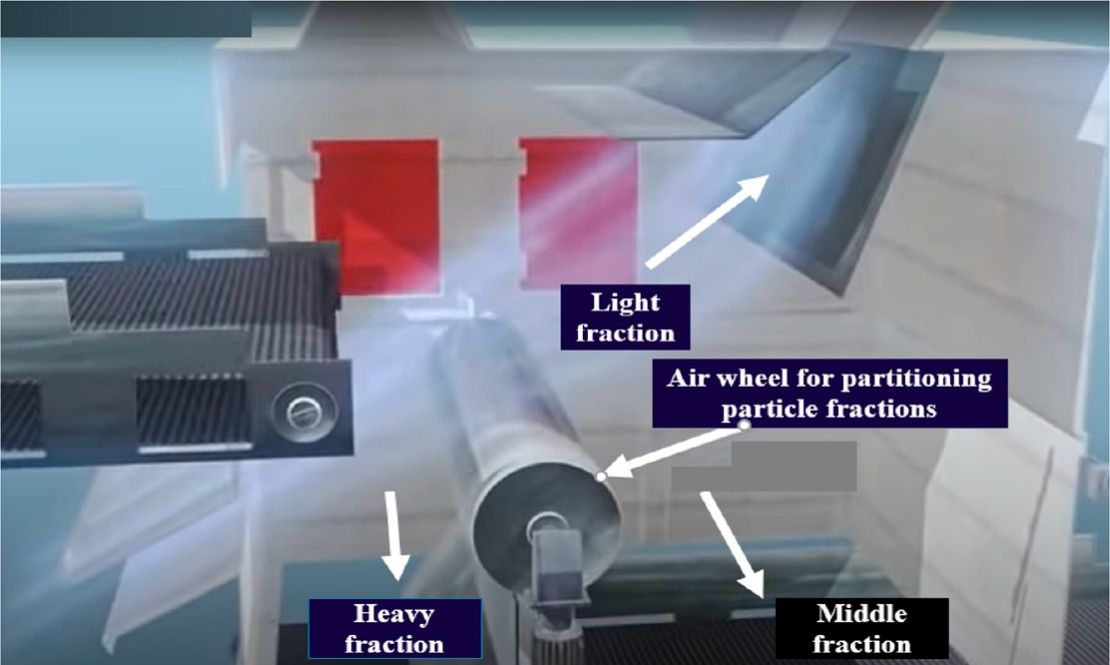
Image showing fractions after classification, Image courtesy of and adapted from Schulz and Berger.^21^

When in operation, the feed material is fed through a conveyor belt with an adjustable feed rate into the already flowing airstream as shown in Figure 13. The heavy fractions as shown in Figure 14 fall into their designated discharge tray/belt, and the same for the middle fraction. The light particles are then sucked upward out of the separation chamber into their own designated collection point.

**Figure 15:**
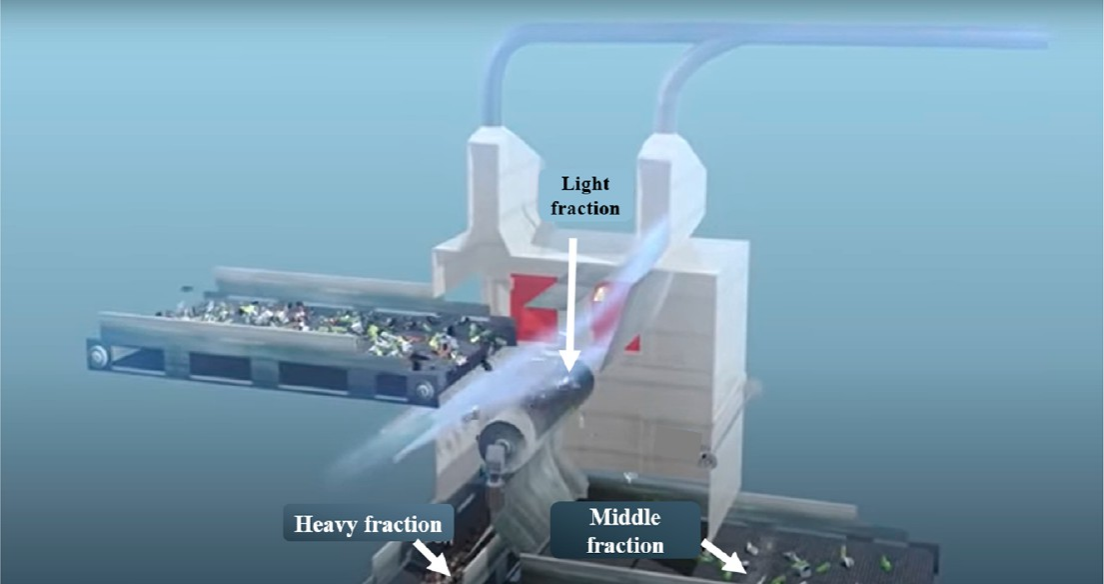
Image showing fractions after classification, Image courtesy of and adapted from Schulz and Berger.^21^

The major function of the air wheel is to partition the air stream carrying the particles into their respective sections. The air wheel is driven by the air stream, which reduces the kinetic energy of the air stream, making the particles being transported attain their terminal velocities. Figure 15 shows the separation process of the 3-fraction wind-sifter separator.

**Figure 16:**
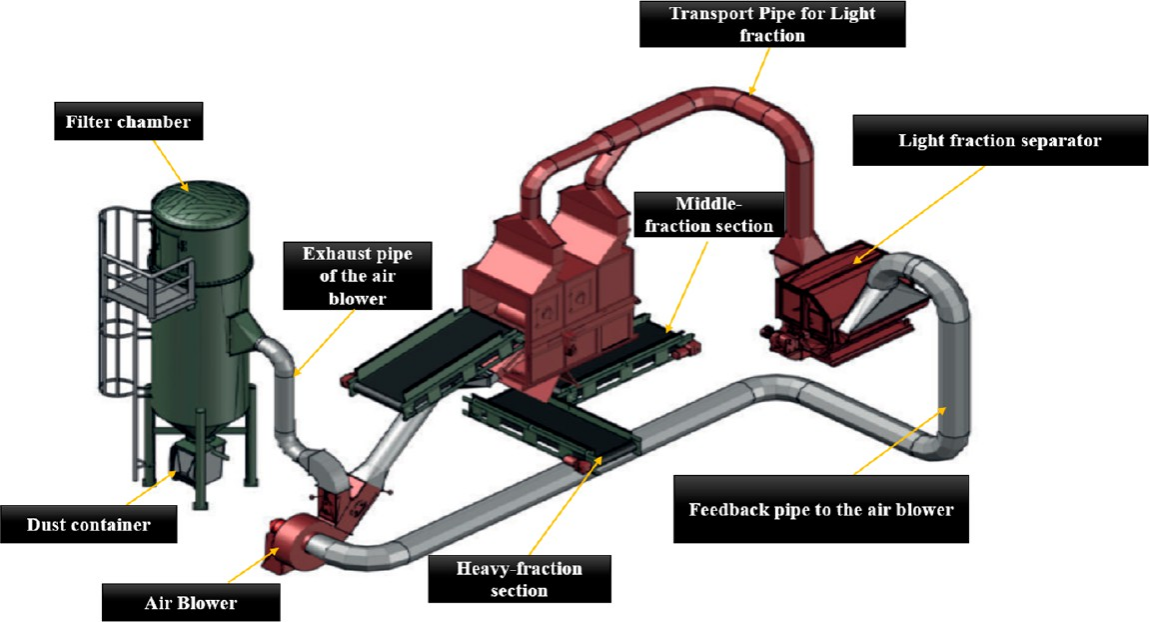
Image showing the 3-fraction separator connected to a light-fraction separator and filter chamber. Image courtesy of and adapted from Schulz and Berger.^21^

**Figure 17:**
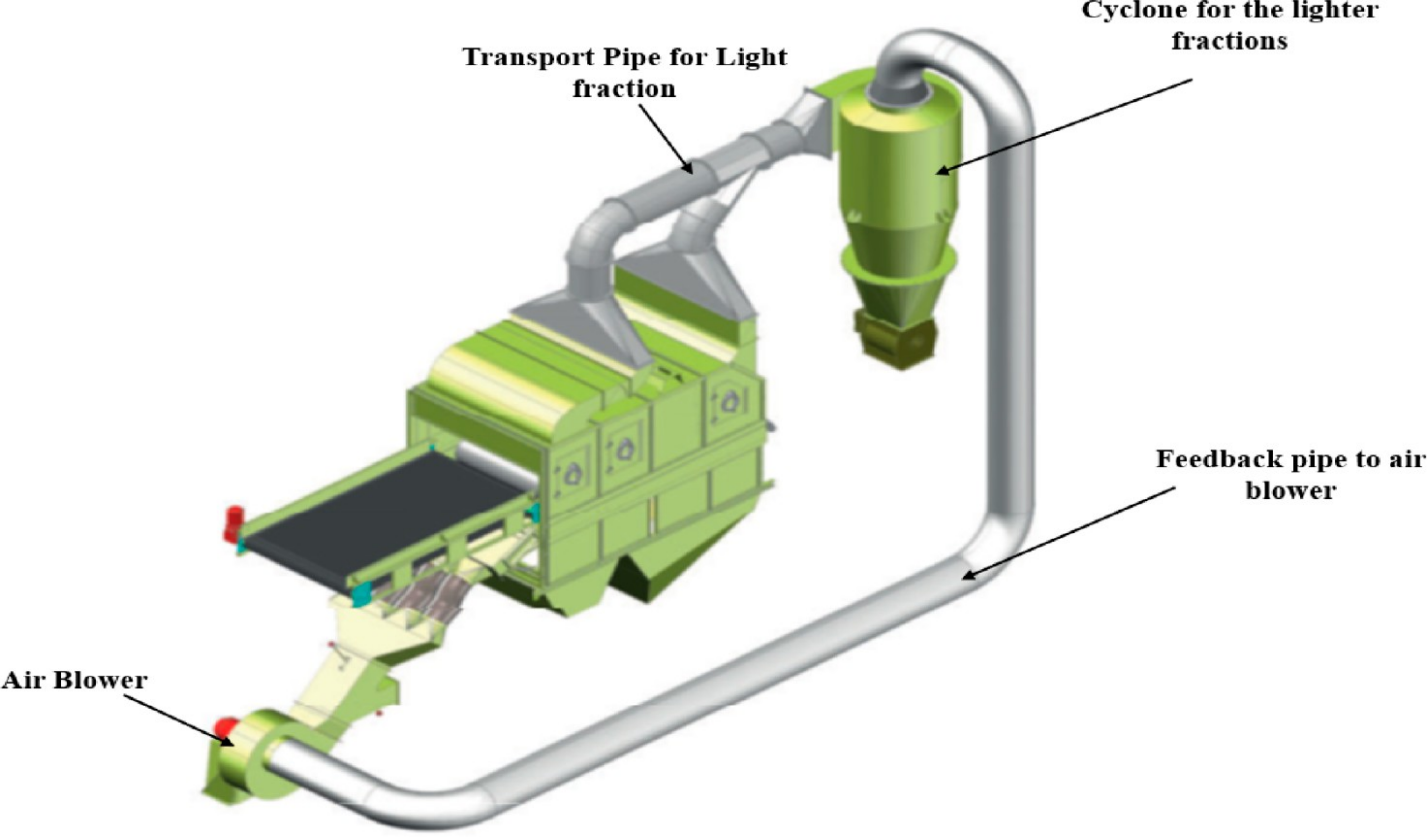
Image showing the 3-fraction separator connected to a gas cyclone. Image courtesy of and adapted from Schulz and Berger.^21^

This type of wind-sifting separator, like the other two, can be a standalone unit or used in tandem with a dust extraction chamber or with a cyclone as can be seen in Figures 16 and 17. This enables the separator to efficiently process the lighter fractions of the separated particles, and the separation operation is usually a closed circuit as can be seen in both figures. Figure 16 shows the total assembly of a 3-fraction wind-sifter separator connected to a light fraction separator and a dust filter chamber. In the light-fraction separator, the light fractions are separated into fine and superfine particles, while Figure 17 shows the 3-fraction separator connected to a gas cyclone for further separation of the stream.

### 2.4 Security Concerns: Health, Safety, and Electrification of Powders during Processing

It must be stated that in the pneumatic processing of fine particles, particularly powdery materials, including coal, some concerns must be outlined. One of these issues is the safety of processing and managing fine/powdery materials. Powdered materials can produce airborne dust containing inhalable particles during processing or handling, which ultimately poses a health hazard to the environment. Some of these health problems range from slight irritation to autoimmune diseases and cancer growth.^22,23^ Another safety problem is the danger of explosion of the combustible powder. The issue of explosion of powders can either be as a result of the powders being highly reactive with the presence of an ample amount of the particle dust cloud^23^ or as a result of electrostatic discharges or mechanically generated sparks.^22,23^ To prevent this, Hoppe et al.^23^ suggested that an inert environment, e.g., a nitrogen atmosphere, must be maintained as this inhibits the availability of oxygen for combustion during the pneumatic transportation of flammable powdery materials.

A second concern in pneumatic processing of powdery/fine particles that must be outlined is the issue of powder electrification. When powdery materials are being pneumatically transported, the particles tend to collide with each other and with the walls of the transport chamber, which then leads to the accumulation of electrostatic charges. This phenomenon is known as triboelectric charging and may result in hazardous spark discharges.^24^ To avert this phenomenon, Nifuku and Katoh^25^ suggested that the vessels for the pneumatic transport of granular/powdery materials be earthed to discharge any charge buildup as well as increasing air velocity to facilitate a diluted phase as it greatly reduces collisions between particles.

## 3 Conclusion and Prospects

Wind-sifter separators have been used for many decades in various fields, particularly in fields like municipal solid waste processing and recycling. They have also been found to be highly appropriate for a variety of applications. This technique has been used in the agricultural to pharmaceutical sectors and even utilized for dry coal beneficiation with a feasibility to be applied to other mineral-processing sectors. The major factors that affect the separation process in wind-sifters are the airflow velocity, mass flow rate, and design of the separators. These factors can be tweaked to suit different applications, that is, in the separation of different materials that include activated carbon, graphite, glass, calcium carbonate, mica, etc.

The wind-sifter separator can also be a standalone unit and can be integrated with other techniques. More investigations are needed to be carried out to understand wind-sifting techniques and to exhaust every possible applicable field to improve its separation efficiency. Computer simulation programs that enable numerical simulations, computational fluid dynamics (CFD), should also be used to understand the impact of process parameters on “particle to wall” and “particle to particle” interactions within the separators.

## References

[ref1] Shapiro M.; Galperin V. Air classification of solid particles: A review. Chem. Eng. Process.: Process Intensif. 2005, 44, 279–285. 10.1016/j.cep.2004.02.022.

[ref2] Alade J.; Bada S. O.; Schmitz W. Computer-Aided Design and Fabrication of a Dry Wind-Sifter Separator. ACS omega. 2021, 6, 20309–20320. 10.1021/acsomega.1c02192. 34395979 PMC8358971

[ref3] Kaas A.; Mütze T.; Peuker U. A. Review on zigzag air classifier. Processes 2022, 10, 764 10.3390/pr10040764.

[ref4] Kuyumcu H. Z.; Rosenkranz J. Investigation of fluff separation from granulated waste plastics to be used in blast furnace operation. 2009. https://www.diva-portal.org/smash/get/diva2:1011223/FULLTEXT01.pdf (accessed June 5, 2023).

[ref5] Mielke C. Ballistic separators for c&d and landfill mining applications: Technical features of heavy duty ballistic separators. 2018. https://www.diva-portal.org/smash/get/diva2:1651639/FULLTEXT01.pdf#page=107 (accessed January 29, 2023).

[ref6] Jang C.; Kahn N.; Langlois L.; Liu R.; Montanaro G. Design of a Winnowing Machine for West African Rice Farmers. 2014. https://escholarship.mcgill.ca/concern/reports/6t053g21q (accessed February 28, 2023).

[ref7] Nishinari K.; Williams P. A.; Phillips G. O. Review of the physico-chemical characteristics and properties of konjac mannan. Food hydrocolloids 1992, 6, 199–222. 10.1016/S0268-005X(09)80360-3.

[ref8] Luijsterburg B.; Goossens H. Assessment of plastic packaging waste: Material origin, methods, properties. Resour., Conserv. Recycl. 2014, 85, 88–97. 10.1016/j.resconrec.2013.10.010.

[ref9] Stebbins A. H. Air classifier.US 1,861,248, 1932. https://patentimages.storage.googleapis.com/d7/9d/bf/e8286c018d2249/US1861248.pdf (accessed November 22, 2022).

[ref10] Roloff C.; Lukas E.; van Wachem B.; Thévenin D. Particle dynamics investigation by means of shadow imaging inside an air separator. Chem. Eng. Sci. 2019, 195, 312–324. 10.1016/j.ces.2018.09.020.

[ref11] Hagemeier T.; Glöckner H.; Roloff C.; Thévenin D.; Tomas J. Simulation of Multi-Stage Particle Classification in a Zigzag Apparatus. Chem. Eng. Technol. 2014, 37, 879–887. 10.1002/ceat.201300670.

[ref12] Mann H.; Roloff C.; Hagemeier T.; Thévenin D.; Tomas J. Model-based experimental data evaluation of separation efficiency of multistage coarse particle classification in a zigzag apparatus. Powder Technol. 2017, 313, 145–160. 10.1016/j.powtec.2017.03.003.

[ref13] Roloff C.; Lukas E.; van Wachem B.; Thévenin D. Particle dynamics investigation by means of shadow imaging inside an air separator. Chem. Eng. Sci. 2019, 195, 312–324. 10.1016/j.ces.2018.09.020.

[ref14] Reddy Y. H.; Purushothaman K.; Sreenivasan M.; Sivakumar E. R. Design and Analysis of Zigzag Classifier in Food Industry Applications. J. Phys.: Conf. Ser. 2021, 2040, 012040 10.1088/1742-6596/2040/1/012040.

[ref15] ERGA Global. Zigzag Air Separators. 2020. https://rusmagnet.com/catalog/gravity-separators/zig-zag-air-separator/ (accessed October 25, 2022).

[ref16] Impact Air Systems. 2021. URL: https://impactairsystems.com/wp-content/uploads/2022/05/Materials-Recovery-Brochure.pdf (accessed October 25, 2022).

[ref17] Trennso-Technik. Wind-Sifter. 2018. URL: https://www.tst.de/en/machines-and-modules/wind-sifter-technology (accessed October 25, 2022).

[ref19] Gericke. Centrifugal Sifters, 2022. https://www.gerickegroup.com/fileadmin/user_upload/Downloads/Brochures/UK/661_4_UK_Gericke_Brochure_Sifter.pdf (accessed December 29, 2022).

[ref18] Shree Bhagwati Group of Companies. Centrifugal Sifters. 2020. URL: https://www.vibrosifter.net/centrifugal+sifters.html (accessed June 29, 2023).

[ref20] Walton O.; Dreyer C.; Riedel E. Centrifugal size-separation sieve for granular materials.US 9,073,088 B2, 2015. https://ntrs.nasa.gov/api/citations/20150014982/downloads/20150014982.pdf (accessed December 29, 2022).

[ref21] Schulz and Berger. 2014. https://www.schulz-berger.com/media/pages/produkte/windsichter/2783218338-1597936937/schulz-berger-broschuere_dt.pdf. (accessed September 14, 2022).

[ref22] Benson J. M. Safety consideration when handling metal powders. J. South. Afr. Inst. Min. Metall. 2012, 112, 563–575.

[ref23] Hoppe T.; Jaeger N.; Terry J. Safe handling of combustible powders during transportation, charging, discharging and storage. J. Loss Prev. Process Ind. 2000, 13, 253–263. 10.1016/S0950-4230(99)00036-4.

[ref24] Ceresiat L.; Grosshans H.; Papalexandris M. V. Powder electrification during pneumatic transport: The role of the particle properties and flow rates. J. Loss Prev. Process Ind. 2019, 58, 60–69. 10.1016/j.jlp.2019.01.010.

[ref25] Nifuku M.; Katoh H. A study on the static electrification of powders during pneumatic transportation and the ignition of dust cloud. Powder Technol. 2003, 135-136, 234–242. 10.1016/S0032-5910(03)00163-3.

